# Case Report: Unlocking arteria Lusoria challenges: sternotomy's role in a single-stage aneurysm repair and artery realignment

**DOI:** 10.3389/fcvm.2025.1557293

**Published:** 2025-04-07

**Authors:** Ziyad Gunga, Lorène Rousseau, Margaux Wolff, Augustin Rigollot, Anna Nowacka, Filip Dulgorov, Zied Ltaief, Valentina Rancati, Rafael Trunfio, Sebastien Déglise, Matthias Kirsch

**Affiliations:** ^1^ Cardiac Surgery Department, Lausanne University Hospital (CHUV), Lausanne, Switzerland; ^2^ Anesthesiology Department, Lausanne University Hospital (CHUV), Lausanne, Switzerland; ^3^ Vascular Surgery Department, Lausanne University Hospital (CHUV), Lausanne, Switzerland

**Keywords:** arteria lusoria, cardiac surgery, vascular, sternotomy, dysphagia case report

## Abstract

Arteria lusoria (AL), an anomaly of the right subclavian artery, occurs in 2% of individuals and can cause symptoms such as dysphagia due to its retroesophageal course. Often associated with Kommerell's diverticulum (KD), a dilation at the artery's origin, this condition poses risks of rupture or dissection. Symptomatic cases and aneurysms necessitate surgical intervention, while asymptomatic cases may warrant observation. We present a case of a 44-year-old woman with dysphagia lusoria due to AL and KD, confirmed by imaging. Given anatomical complexities, a one-stage open surgical repair via sternotomy was performed. This involved resecting the KD and creating a neo-trajectory for the right subclavian artery using a Dacron tube graft. Cardiopulmonary bypass ensured safe manipulation, and post-operative imaging confirmed excellent outcomes, with complete symptom resolution. Surgical approaches for AL and KD vary depending on anatomy and symptomatology, ranging from open repairs to hybrid and endovascular techniques. Open thoracotomy remains the gold standard for young patients without comorbidities. Hybrid approaches are reserved for emergencies or high-risk patients, offering reduced morbidity but potentially higher complication rates. Advances in imaging and surgical techniques, including hybrid methods, have improved outcomes, with mortality rates significantly lower than historical benchmarks. The 2024 EACTS/STS guidelines recommend open surgery for young, fit patients (Class I, Level C) and hybrid approaches for emergencies or patients unfit for open surgery (Class I, Level C). Our case exemplifies the feasibility of sternotomy in providing precise, effective correction for KD with AL in a single operation while minimizing risks associated with other approaches.

## Introduction

The development of the aortic arch and its branches during the early stages of fetal life provide an important vascularization network (1). However, some malformations can occur and lead to a disturbed arterial path, which can be symptomatic during adult life. Arteria lusoria (AL) is the most common malformation with an incidence of 2% and affects the right subclavian artery (2). Under physiological conditions, the right subclavian artery arises together with the carotid artery from the brachiocephalic trunk, a branch of the aortic arch (1). In patients with AL, the subclavian artery arises directly from the aortic arch and reaches the arm with a sinuous trajectory. The aberrant right subclavian artery often crosses behind the esophagus, or occasionally between the esophagus and the trachea. Additionally, in 60% of the patients, the origin of this artery leads to a dilatation of the aorta at the level of its emergence (3). This condition, called Kommerell's diverticulum (KD), is usually symptomatic and generally manifested by late-onset dysphagia (3, 4). The surgical treatment of this malformation is determined by two criteria: the AL must be either symptomatic or it must be combined with an aneurysm (5). Numerous techniques have been proposed for addressing these malformations, yet a definitive consensus on the most efficacious method remains elusive. Here in our study, we propose an existing approach aimed at simultaneously repairing the aneurysm and realigning the artery to its proper course in a single procedure, especially in cases where endovascular treatment is not anatomically feasible.

## Case report

**Figure 1:**
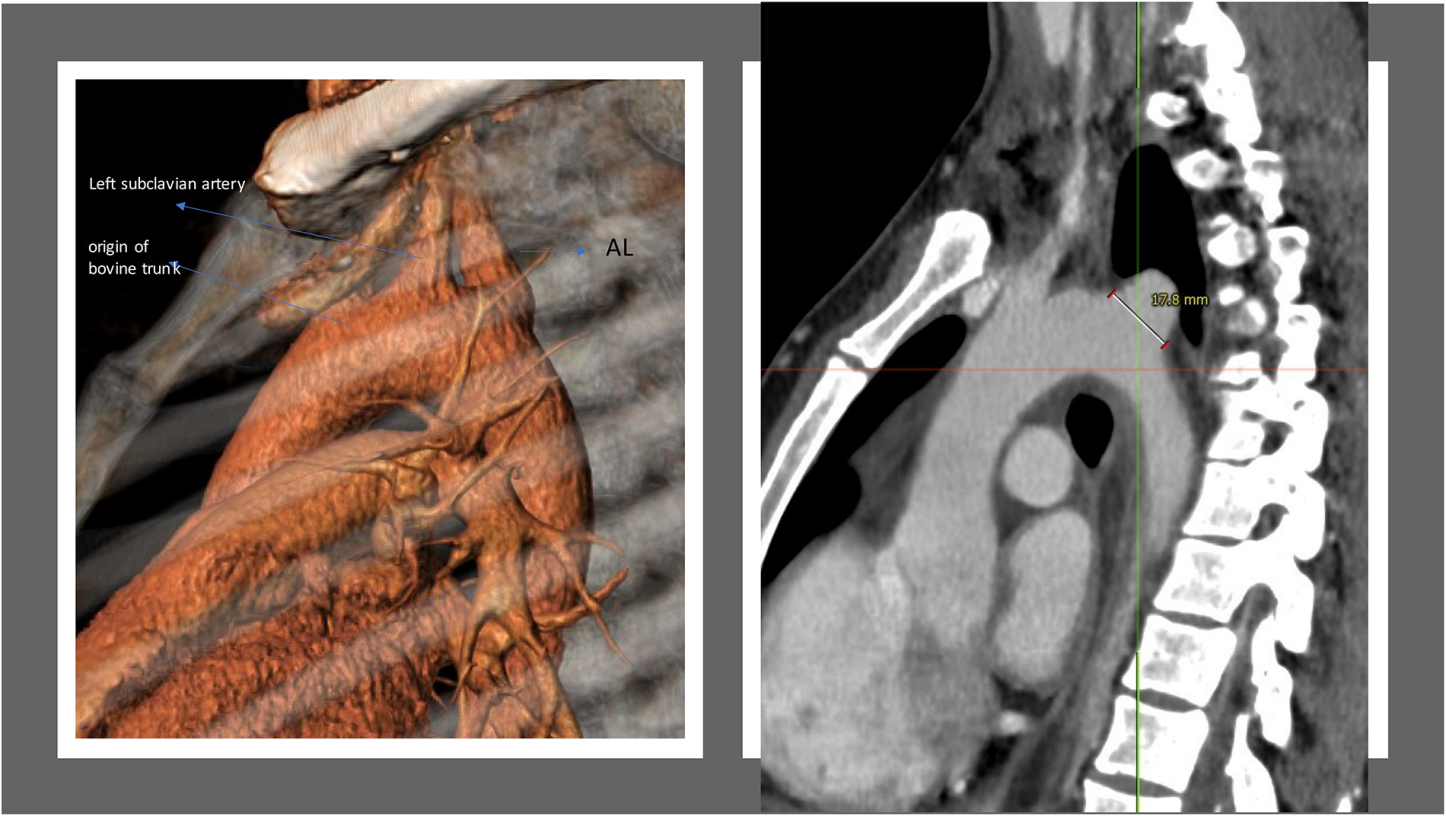
3-Dimension reconstruction and sagittal CT scan view revealing an aberrant origin of the right subclavian artery from the aortic arch and a dilated appearance of its proximal segment (17.8 mm), with a retro-esophageal course.

**Figure 2:**
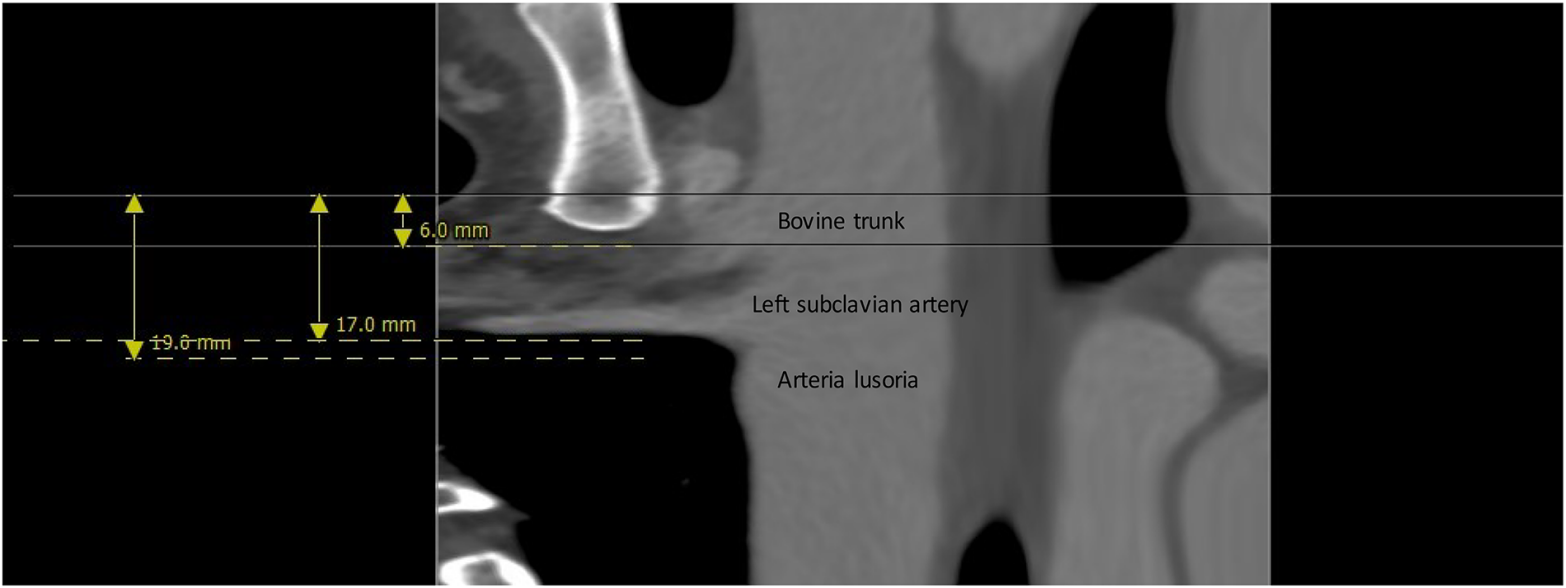
Center line reconstruction imaging by 3mensio medical imaging (utrechtThe Netherlands): It underscores the remarkably proximity between the arteria lusoria and the bovine trunk, measuring a mere 13 mm, alongside the minimal 2 mm separation between the AL and the left subclavian artery.

A 44-year-old patient underwent gastric bypass surgery in 2015, resulting in a remarkable weight loss of 30 kg. The patient presented with a progressively worsening dysphagia over the past six months. Diagnostic investigations revealed a pulsatile compression of the esophagus, suggestive of dysphagia lusoria. Subsequent computed tomography (CT) imaging confirmed the presence an aberrant origin of the right subclavian artery from the aortic arch which exhibits ectasia in its proximal portion (Kommerell diverticulum), with a diameter reaching up to 17,8 mm and a retroesophageal course causing compression on the posterior wall of the esophagus. Additionally, there is a presence of a bovine bicarotid trunk, with a 17 mm distance separating it from the subclavian artery. Centerline reconstruction emphasized a narrow 2 mm space between the arteria lusoria and the left subclavian artery (Figures 1, 2). Given the symptomatic nature of the condition and the concurrent presence of Kommerell's diverticulum, surgical intervention was deemed necessary. However, due to the close proximity of the bovine trunk and the arteria lusoria, treatment by TEVAR or hybrid open surgery was considered tedious and relatively at risk.

The selected surgical approach involved a median sternotomy, enabling a one-stage procedure involving resection of Kommerell's diverticulum and the establishment of a neo-trajectory. Alternatively, the thoracotomy approach was a viable choice, albeit entailing a two-stage procedure. This neo-trajectory involved the reinsertion of the right subclavian artery into the ascending aorta, achieved through the interposition of a 10 mm Dacron tube. The surgical process commenced with an upper laterotracheal dissection to expose the aberrant right subclavian artery, located retro esophageally. The origin of the artery was demarcated by the presence of the Kommerell's diverticulum. Following the establishment of cardiopulmonary bypass (CPB) between the ascending aorta and an atrio-caval cannula, the distal portion of the right subclavian artery was clamped at its origin, and the arteria lusoria, along with its dystrophic segment, was excised.

**Figure 3:**
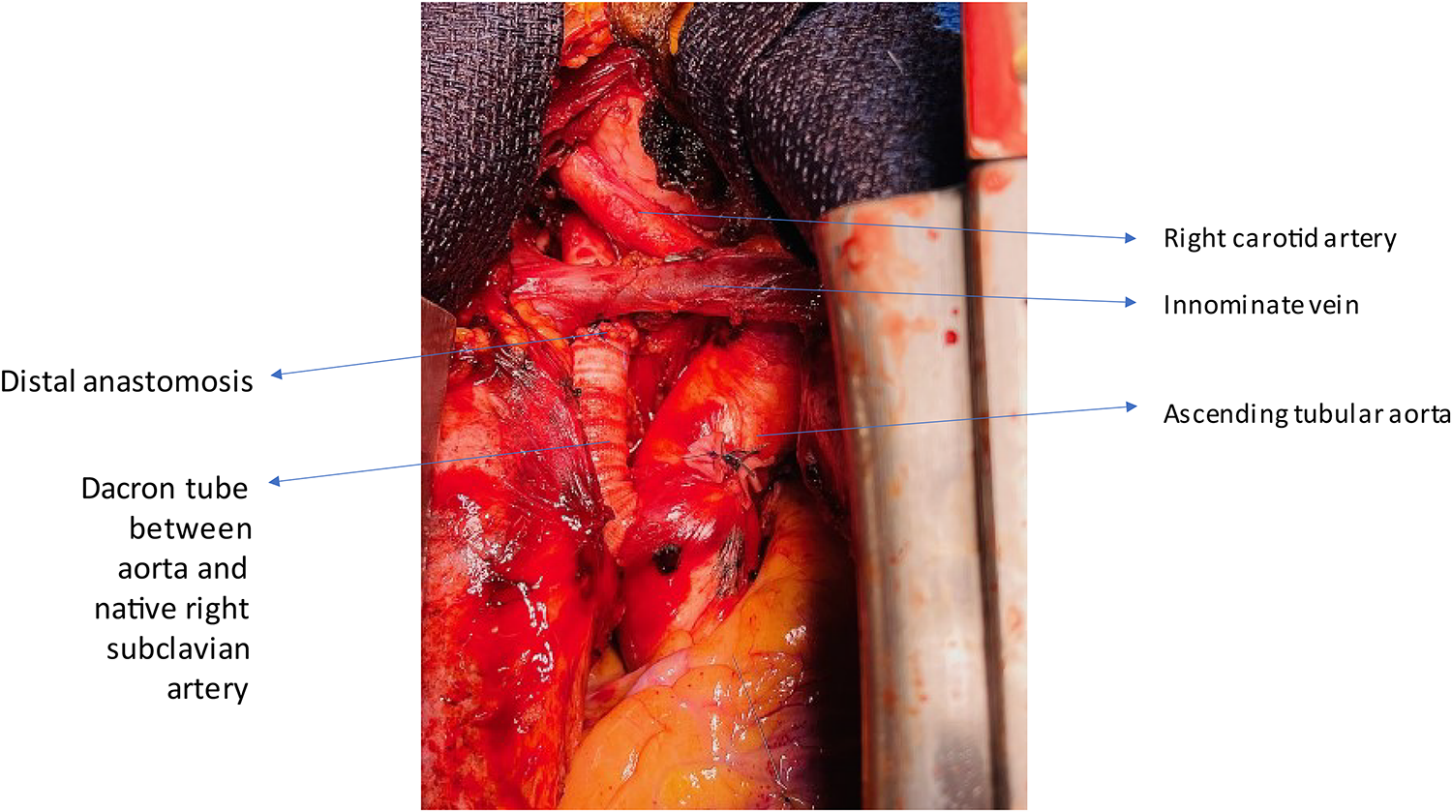
Operative finding showing the interposition of a dacron tube to connect the ascending aorta to the right subclavian artery.

**Figure 4:**
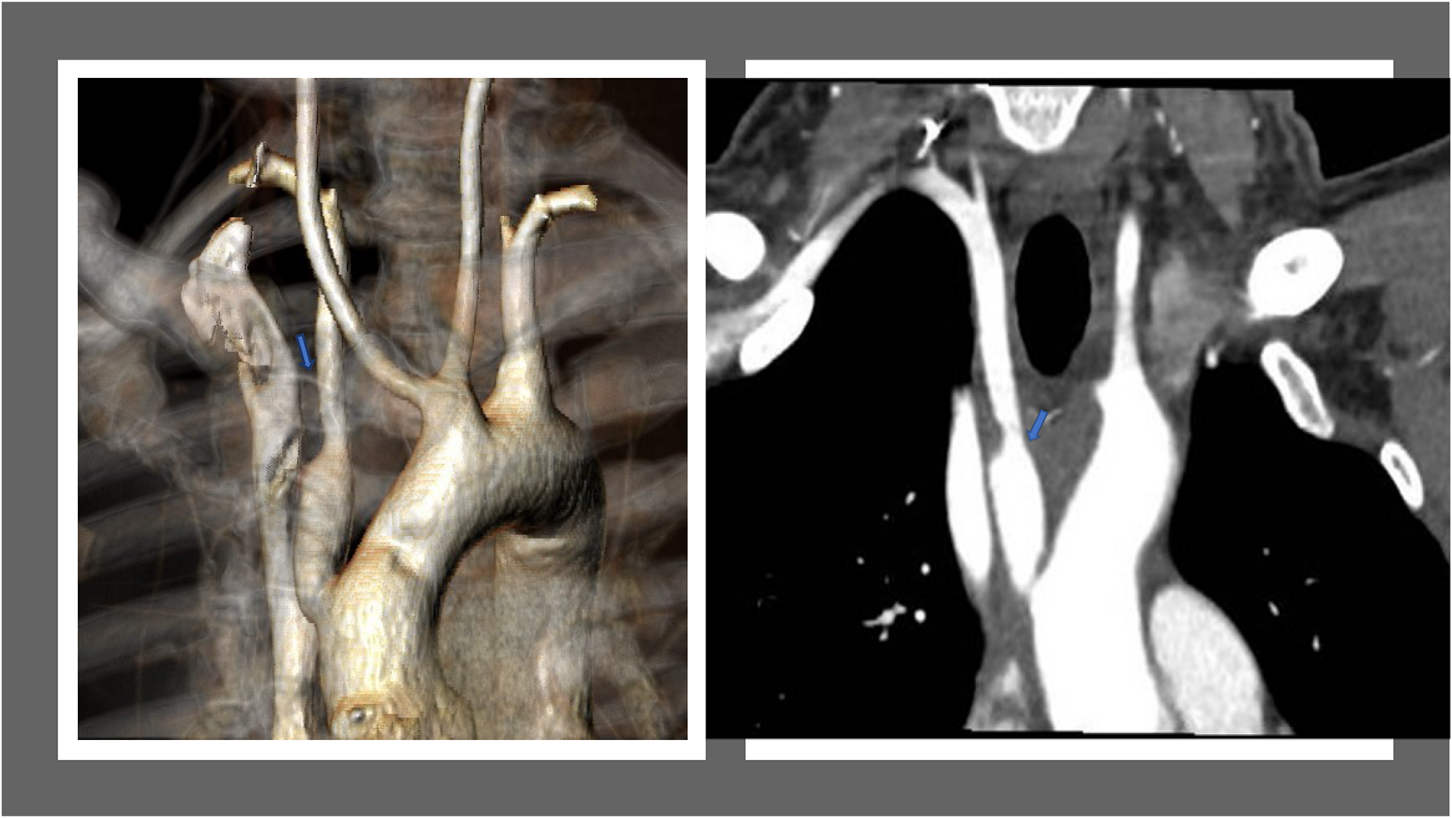
(left)post-operative three-dimensional CT reconstruction imaging depicts the newly established pathway of the right subclavian artery. (right) 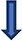 An arteriography highlighting a normal right subclavian route.

Closure of the resection site was meticulously executed through a double suture technique, fortified by the application of two extra-luminal pericardial pledgets. Temporary lateral aortic clamping, applied anterolaterally, facilitated the successful interposition of a 10 mm Dacron prosthesis between the aorta and the right subclavian artery, positioned beneath the innominate vein (Figure 3). CPB facilitates the safe manipulation of the heart and aorta without compromising hemodynamics, thereby enabling the secure excision of the dystrophic segment. Its utilization is not mandatory and can be tailored according to the complexity of the anatomy. Intra-operative assessments affirmed the prosthesis's excellent patency, along with secure anastomotic sealing. Subsequent post-operative follow-up (Figure 4) revealed an uneventful recovery, and she was relieved of dysphagia.

## Discussion

The aortic arch and its branches can exhibit anatomical variations that have been described since the first half of the 18th century by anatomists such as Hummel and Hunauld (6). These anomalies share a common embryological origin, giving rise to diverse clinical presentations. Some anomalies remain asymptomatic and are discovered incidentally, while others can lead to severe clinical symptoms from birth. The most common anomaly involving the branches of the aortic arch pertains to the right subclavian artery. Instead of arising jointly with the right carotid artery to form the brachiocephalic trunk, it originates directly from the aorta downstream from the left subclavian artery. It then follows an aberrant course to supply the right upper limb. This artery was aptly named Arteria Lusoria, or the “jesting artery,” in 1794 (7).

Surgical intervention for arteria lusoria and its associated pathologies hinges on specific clinical contexts. For asymptomatic, non-aneurysmal arteria lusoria, there is no strict indication for surgery unless it is associated with adjacent aortic pathology. However, symptomatic presentations, such as dysphagia, upper limb ischemia, or vertebral-basilar territory ischemia, necessitate surgical correction. Similarly, aneurysmal aberrant right subclavian arteries (ARSA) require intervention regardless of symptomatology due to their elevated risk profile. Kommerell diverticulum (KD), frequently associated with ARSA, emerges as a critical finding in 60% to 82% of cases. KD is characterized by an aneurysmal dilation at the origin of the aberrant artery within the descending aorta, posing a considerable risk of dissection or rupture with incidences ranging from 19% to 53% (8). Backer et al. recommend surgical intervention when the aberrant artery diameter exceeds 1.5 times that of the anatomic subclavian artery. Additionally, repair is warranted for aneurysms demonstrating rapid growth exceeding 5 mm within six months (9). Surgery for symptomatic KD is a well-established indication, irrespective of size or growth, given the significant risks of rupture and dissection. These complex interventions are associated with low periprocedural mortality rates. In contrast, prophylactic surgical recommendations for asymptomatic patients rely on limited data from single-center reports and multicentric observational registries, leading to variability in guidelines and a generally low level of evidence. Observation is typically deemed appropriate for asymptomatic KD and ASCA. However, preventive surgery is advocated when the surgical risk is demonstrably lower than the risk of rupture or dissection. Consensus statements from the EACTS/European Society for Vascular Surgery suggest surgery for subclavian arteries exceeding 30 mm in diameter and KD larger than 55 mm (10). Similarly, the American College of Cardiology/American Heart Association guidelines (11) align with the 2020 SVS clinical practice recommendations (12), advocating intervention for orifices exceeding 30 mm or diverticula larger than 50 mm. A multicenter registry study by Bath et al., encompassing 285 patients, underscores worse outcomes in symptomatic cases, a significant inherent rupture risk in untreated KD, and a comparably low risk of intervention (13).

Several technical considerations confront surgeons when addressing arteria lusoria. Firstly, the selection of the surgical approach is pivotal (Table 1), given the deep vascular location of the lusoria artery in the mid-posterior mediastinum, oriented from bottom to top and left to right. This variability has historically led to various surgical approaches, including left or right thoracotomy, median sternotomy, low tie-neck cervical incision, and right supraclavicular cervical incision. Hybrid open and endovascular techniques, integrating proximal thoracic endovascular aortic repair (TEVAR) with a combination of extra-anatomic cervical artery debranching or supra-aortic trunk vessel stenting, have become more illustrious than the traditional open techniques. This surge in popularity is driven by the promising prospect of decreased morbidity associated with these approaches. Secondly, the other prominent technical consideration is the restoration of arterial continuity in the right upper limb. The selected surgical strategy primarily hinges on whether the aberrant right subclavian artery is aneurysmal or not, as well as the presence of associated lesions in the adjacent aorta. Additionally, Settembre et al. have established morphometric and anatomical criteria facilitating the feasibility of endovascular treatment (14). Ensuring a neck length of at least 20 mm is crucial for proper apposition in the thoracic aorta. However, accurately measuring the distance between the two subclavian arteries presents challenges due to the complex three-dimensional configuration of the aortic arch, unless assisted by three-dimensional volumetric reconstructions, and manual centerline reconstruction techniques.

**Table 1:** The different surgical approaches to treat arteria Lusoria.

Surgical approach	Pros	Cons	Possibilities
Full median sternotomy	− Provides excellent exposure of the aorta on both sides of the lesions	− Invasive	− Allows direct control over the origin
	− Preserves and visualizes the recurrent nerve on the right	− Scar of 20 cm	− Direct access to treat aneurysmal lesions in one step surgery
	− Facilitates direct access to treat aneurysmal lesions − Onestep surgery with the possibility to clamp the aorta		
Right supraclavicular	− Provides excellent exposure of the esophageal path	− Risk of arterioesophageal fistulae	− Can be combined with hybrid endovascular approach
	− Minimally invasive	− No control of the origin	
	− Provides easier access for freeing the artery from the esophagus	− Necessity to combine with other approaches to treat vascular lesions − Potential for injury to surrounding structures	
Thoracotomy	− Provides excellent exposure of the aorta	− Invasive	− Allows access for proximal and distal ligation of artery
	− Facilitates direct access for treating aneurysmal lesions	− Difficult to control in case of vascular injuries	− Direct access for treating aneurysmal lesions
		− Twostage	
		− Painful	
Supraclavicular cervical	− Provides excellent exposure of the esophageal path	− No control of the origin	− Ideal for treating dysphagia
	− Facilitates easier access for removing the artery from the esophagus	− No possibilities to treat vascular lesions − Potential for injury to surrounding structures	
TEVAR	− Minimally invasive	− May not fully address all anatomical variations and complications associated with arteria lusoria	− Potential to mitigate perioperative risks associated with open surgeries
	− No scar − Provides an option for highrisk patients who are not suitable candidates for open surgery	− Requires expertise in endovascular techniques − Risk of endoleak or migration of the stent graft	− Can be combined with other surgical approaches for comprehensive treatment of arteria lusoria

In addressing non-aneurysmal aberrant right subclavian arteries, the isolated cervical route stands out as the foremost preferred open surgical approach. This technique involves a supraclavicular cervicotomy on the side of the arteria lusoria, providing access to the artery's pre-scalene segment through an inter-jugulo-carotid pathway. The esophagus is carefully repositioned forward after releasing it from fibrous attachments that secure it to the spine. This maneuver facilitates continued dissection towards the artery's aortic origin, allowing for ligature under direct visual control. A posterolateral left thoracotomy is commonly considered the approach of choice in the presence of KD as described in the series of Loschi et al. (15) as well as the registry of Bath et al. (13), as it enables adequate aortic control on both sides of the aneurysm. However, full lateral clamping around the origin of a lusoria aneurysm may not always be feasible, prompting the need for complete aortic clamping to facilitate lesion repair. While a simple closure of the aneurysmal neck with a prosthetic patch may suffice in certain instances, the presence of concomitant aortic lesions may necessitate partial prosthetic replacement of the descending thoracic aorta.

In complex scenarios or when a more precise control of the artery's origin is required, the possibility of employing median sternotomy presents itself. We advocate for this approach as it provides safety measures and meticulous control facilitated by the extracorporeal circulation machine. It's important to note that while this approach is notably invasive, it generally entails less post-operative discomfort when compared to the left thoracotomy technique. It can be associated with recurrent nerve injury, chyle leak and phrenic nerve injury and sternitis.

With the advent of less invasive endovascular techniques, there has been a surge in exploring alternative strategies, hence, reducing the need for traditional open surgeries. This trend is underscored by heightened enthusiasm in mitigating perioperative invasiveness. The widespread adoption of thoracic endovascular aortic repair (TEVAR) has revolutionized the management of complex thoracic and aortic arch diseases, offering promising avenues to address complications associated with aortic arch syndrome. Notably, Czerny et al. (10) have reported favorable mid-term outcomes with supra-aortic transpositions for extended endovascular repair of aortic arch aneurysms. In 1998, Davidian et al. (16) pioneered the description of an exclusion procedure for an AL aneurysm using a covered stent. Scant literature exists on the utilization of occlusion systems in AL (17) in conjunction with a right carotid-subclavian bypass, with the risk of migration. Only a sparse number of series detailing the management of Arteria Lusoria using hybrid techniques have been reported, and purely endovascular treatment has been performed in a limited subset of cases (18).

The imperative for cervical vessel revascularization alongside endograft coverage has spurred the development of enhanced hybrid open and TEVAR reconstruction techniques. These encompass innovative approaches such as TEVAR with extra-anatomic bypass options like carotid-subclavian bypass or transposition, TEVAR integrated with cervical vessel fenestration or chimney techniques, and the strategic utilization of partial aortic arch debranching in conjunction with TEVAR. Considering the proximity observed between the aberrant subclavian artery and the left subclavian artery, as indicated by the radiographic assessment conducted by Settembre et al. (14), revealing an average distance of 5 mm, bilateral subclavian artery coverage is frequently unavoidable. Indeed, Baker et al. and Tinelli et al. have documented favorable outcomes with hybrid techniques (9, 19). Nevertheless, various studies on the hybrid approach have highlighted several complications, including perioperative mortality, nerve injury, stroke, spinal cord ischemia, and endoleak. The reported incidence of major adverse events ranges from 0% to 23%. Gray et al. has highlighted a significant endoleak rate in their series reaching 44% with a technical success rate of 83% (20).

An in-depth analysis of existing literature before 2020 underscores that open surgical interventions aimed at arteria lusoria aneurysms have historically carried mortality rates ranging from 25% to 50%. It's noteworthy that many of the cited studies are dated, and ongoing advancements in techniques and materials have substantially improved both in safety and effectiveness. A recent systematic review conducted by Loschi et al. (15) in 2023 revealed encouraging findings regarding the 30-day mortality rates following interventions for arteria lusoria with concomitant KD. The study found low mortality rates for open repair (3.5%), hybrid (6.8%), and endovascular (3.9%) approaches. The risk of stroke was also relatively low for open (4.9%) and hybrid (4.1%) procedures, with slightly higher rates associated with endovascular repair (9.8%). These results suggest that all three surgical modalities demonstrate favorable safety profiles and effectiveness in achieving appropriate outcomes during both immediate and mid-term postoperative periods. Therefore, we firmly believe that the exclusion of the Kommerell diverticulum, coupled with the reimplantation of the aberrant vessel through a newly designed and anatomically precise trajectory branched to the aorta via a sternotomy access, provides a unique and more precise correction. However, this advancement does entail an associated morbidity which is inherent to contemporary elective open aortic surgery. The EACTS/STS guidelines 2024 (21) recommend open surgical treatment, such as thoracotomy with carotid-to-subclavian artery bypass, as the first-line approach for young patients with Kommerell's Diverticulum (KD) who have no significant comorbidities (Class I, Level C). However, in emergency cases or when patients are unfit for open surgery, a hybrid approach using closed-chest repair is advised as a safe and effective alternative (Class I, Level C). The natural progression of KD is not well established. However, existing studies suggest a highly variable risk of rupture or dissection at the time of diagnosis, with reported rates ranging from 0% to 50%. In a cohort of 32 patients, a significant rupture rate of 19% was observed, with affected diverticula measuring between 4 and 10 cm. Another review focusing on right aortic arch cases found a 6% rupture rate, while combined rupture and dissection occurred in 44% of cases, with diverticulum sizes ranging from 2 to 8 cm. Single-center analyses have also highlighted substantial risks, with one study identifying dissection in 20% (22) of patients and another reporting a dissection rate as high as 50% (23), particularly in cases where the mean diverticulum size exceeded 5 cm. These findings highlight the significant risks associated with Kommerell's diverticulum and emphasize the necessity of vigilant monitoring through Magnetic Resonance Imaging (MRI), which offers the advantage of reduced radiation exposure compared to CT scans. Like the surveillance strategies employed for aortic aneurysms, annual imaging is recommended to track changes in diverticulum size. If a substantial increase in diameter is observed over the course of a year or if the diverticulum exceeds 55 mm, prompt surgical intervention should be considered to mitigate the risk of rupture or dissection (10).

In pediatric patients, a right thoracotomy with a muscle-sparing technique or a sternotomy provides the most effective mediastinal exposure for treating aberrant subclavian artery lesions (AL). These approaches allow optimal mobilization of the distal right subclavian artery and facilitate direct anastomosis to the ipsilateral carotid artery without the need for graft interposition. In contrast, alternative techniques: such as a left thoracotomy combined with a cervical incision or an extrathoracic approach,present notable challenges, including limited vessel exposure, increased difficulty in hemorrhage control, and the need for patient repositioning due to multiple incisions. Similarly, the supraclavicular approach offers suboptimal visualization and heightens the risk of hemorrhagic complications. Furthermore, inadequate exposure may result in a persistent arterial stump behind the esophagus, potentially leading to ongoing symptoms, embolization, or aneurysmal dilatation. Backer et al. (23) proposed an alternative strategy for pediatric patients, advocating for resection of KD, division of the ligamentum, and translocation of the subclavian artery via a left thoracotomy with partial clamping of the descending aorta, regardless of aortic arch laterality. While this approach has been widely utilized, a right thoracotomy or sternotomy remains the preferred option in many cases, as it provides superior access, enhances surgical precision, and optimizes long-term outcomes.

## Conclusion

Our experience underscores that median sternotomy facilitates the concurrent resolution of aneurysmatic lesions and the repositioning of the pathological subclavian artery within a single surgical in a safe and controlled manner. Undoubtedly, Loshi's systematic review (15) has overhauled the existing literature (24, 25) by emphasizing the comparable safety and efficacy of various management strategies for AL with KD, yielding satisfactory early and midterm outcomes. Hence, tailoring treatment to individual patients, considering anatomical nuances and surgical proficiency with an endovascular competence, is paramount to optimize the best patient outcomes.

## Data Availability

The raw data supporting the conclusions of this article will be made available by the authors, without undue reservation.
